# Financial management of large, multi-center trials in a challenging funding milieu

**DOI:** 10.1186/s13063-018-2638-z

**Published:** 2018-05-03

**Authors:** Olivia Lovegreen, Danielle Riggs, Myrlene A. Staten, Patricia Sheehan, Anastassios G. Pittas

**Affiliations:** 1 Research Administration, Department of Medicine, Tufts Medical Center, 800 Washington St, Boston, MA 02111 USA; 2 Kelly Government Solutions for National Institute of Diabetes and Digestive and Kidney Diseases, 900 Rockville Pike, Bethesda, MD 20892 USA; 3 Division of Endocrinology, Diabetes and Metabolism, Department of Medicine, Tufts Medical Center, 800 Washington St, Boston, MA 02111 USA

**Keywords:** Clinical trial, Diabetes, Budget, Hybrid, Financial management, National Institutes of Health

## Abstract

**Background:**

Randomized clinical trials that have public health implications but no or low potential for commercial gain are predominantly funded by governmental (e.g., National Institutes of Health (NIH)) and not-for-profit organizations. Our objective was to develop an alternative clinical trial site funding model for judicious allocation of declining public research funds.

**Methods:**

In the Vitamin D and Type 2 Diabetes (D2d) study, an NIH-supported, large clinical trial testing the effect of vitamin D supplementation on incident diabetes in 2423 participants at high risk for diabetes, a hybrid financial management model for supporting collaborating clinical sites was developed and applied. The funding model employed two reimbursement components: Core (for study start-up and partial efforts throughout the study, ~40% of the total site budget), invoiced by sites, and Performance-Based Payments (for successful enrollment of participants and completion of follow-up visits, ~60% of the total site budget), automatically issued to the sites by the Coordinating Center based on actual recruitment and visits conducted. Underperforming sites transitioned to Performance-Based Payments only.

**Results:**

Recruitment occurred from October 2013 through December 2016, requiring one additional year than the 2-year projection. Median enrollment at each site was 88 participants (range 29–318; 20 to 205% of the site target). At the end of year 1, study-wide recruitment was at 12% of the target (vs. 50% projected) and 12% of the total grant award was invested. The model constantly evaluated sites’ needs and re-allocated resources to meet the study enrollment goal. If D2d had issued cost reimbursement subaward agreements and sites invoiced for their entire budget, 83% of the award would have been spent for all study activities over the first 4 years of the trial compared to 65% of the award spent (US$26M) under the hybrid model used by D2d.

**Conclusions:**

It is feasible to foster a hybrid financial management approach to steward limited available public funds for research in a dynamic and consistent way that does not compromise the trial’s scientific integrity and ensures conservation of funds to complete recruitment and continue to follow up participants.

## Background

Randomized clinical trials are at the top of the hierarchy of research designs and provide the highest level of evidence to advance clinical care. One of the major barriers to undertaking clinical trials is expense. In 2013, the estimated average per-participant cost in industry-sponsored trials was US$36,500 [1]. Trials that address clinically important questions but have no or low potential for financial gain (e.g., study of rare diseases, non-patentable therapies, dietary supplements) are predominantly funded by not-for-profit or governmental agencies, the largest of which in the USA is the National Institutes of Health (NIH). Although the importance of clinical trials is well recognized by the NIH [2], funding is becoming increasingly challenging. From 1994 to 2003, the NIH budget more than doubled from US$11.0B to US$27.1B with a further gradual increase until 2010 when the NIH budget started to decline [3]. However, after accounting for inflation, the NIH budget has declined every year since 2003 and recent proposals by the White House recommend roughly 18% reduction in the NIH budget [4]. Therefore, alternate methods aiming to reduce the costs of large randomized trials, supported by the NIH and other governmental agencies and not-for-profit organizations, are needed.

In large, multi-center clinical trials, the proportion that is allotted to collaborating clinical sites to recruit and retain participants is the largest and most variable component of the budget. Large, multi-center clinical trials have historically employed two models to manage clinical site budgets. The cost reimbursement model provides subaward agreements that cover amounts for personnel effort (i.e., site investigators and staff) and participant expenses (e.g., recruitment, testing, follow-up visits). Under this approach, the subaward amounts are pre-determined based on the site’s projected enrollment and a site could be reimbursed for all expenses despite enrolling few participants. Under this model, if efforts are not promptly undertaken to reduce the costs of underperforming sites, a large proportion of the grant award could be depleted prior to the trial reaching the targeted enrollment, reducing funds for the remaining trial activities. The second model (“performance-based only”) provides a pre-determined reimbursement amount per participant enrolled. This approach conserves funds during slow recruitment and is results-oriented (e.g., focusing on enrollment metrics) but requires sites to “front” the costs for study start-up and initial recruitment efforts. Additionally, this model may not fully consider the variability in regional costs that make research more expensive to conduct in certain high-cost areas [5]. Under this model, the risk of non-performance rests with the clinical site and the amount of anticipated payments is unknown, which makes recipient institutions apprehensive [5].

In the setting of declining public budgets for research, many groups within the USA are exploring alternative approaches to reduce the costs of clinical trials [1]. We describe a hybrid financial management model for collaborating clinical sites, which we applied in the Vitamin D and Type 2 Diabetes (D2d) study, an NIH-supported, large clinical trial testing the safety and efficacy of vitamin D supplementation on the development of diabetes in people at high risk for diabetes [6].

## Methods

### Overview of the study design

D2d is a US-based, multi-center, randomized (1:1), double-blind, placebo-controlled, parallel-group, primary prevention clinical trial comparing oral administration of 4000 IU/day of cholecalciferol (vitamin D_3_) vs. placebo in people at high risk for diabetes who are followed for incident diabetes (primary outcome) for approximately 3 years after randomization (www.d2dstudy.org). Cancer and cardiovascular disease are key secondary outcomes. The design of D2d has been published [6]. The study is approved and monitored by an independent data and safety monitoring board and the institutional review board of each collaborating clinical research site. Target participants were adults at high risk for diabetes, defined as meeting at least two of three glycemic criteria for prediabetes established by the American Diabetes Association in 2010 [7]. Following randomization, participants were seen twice a year and had laboratory testing for ascertainment of diabetes.

D2d is being conducted at 22 US collaborating clinical sites (www.d2dstudy.org/sites) selected by the D2d Coordinating Center and the funding agency because of their ability to recruit and retain a diverse population of people at risk for diabetes. Potential sites submitted applications that detailed past research experience with emphasis on participant recruitment and retention, and provided information on access to potential participants and a D2d-specific recruitment plan. Applications were reviewed by the planning committee and objectively scored. The site budget was not a factor in the selection process. Each site was expected to enroll approximately 100 to 150 participants towards the study-wide enrollment target of 2382. D2d is an event-driven trial that will continue until the required number of diabetes outcome events is reached, which is expected to occur in late 2018.

### Overview of the financial management model

The hybrid financial management model utilized by D2d has two reimbursement components for the sites, Core and Performance-Based Payments (PBPs). Core is provided primarily for study start-up (e.g., institutional review board (IRB) submission, staff training) and partial effort throughout the study for personnel-based activities (recruitment planning, pre-screening, participation in committees). Performance-Based Payments are provided quarterly for successful enrollment of participants and completion of follow-up visits in the preceding 3 months.

### Budget year 1: determination of Core vs. PBP value

At the time of the grant application, each site developed a standard 5-year NIH budget based on the targeted number of enrolled (i.e., randomized) participants at the site. The D2d Coordinating Center provided guidance on personnel effort for a typical site (Table 1). Each site’s approved 5-year budget was used to determine site-specific Core and PBP amounts. At the start of the study, Core was defined as 50% of annual personnel expenses. Each site invoiced D2d quarterly for Core support; the remaining 50% of personnel effort and all non-personnel expenses were recovered through PBPs. PBPs were calculated by deducting the projected value of the Core (over the 5 years) from the approved 5-year budget and dividing the balance by the projected site enrollment to establish a site-specific PBP amount per enrolled participant (Table 2). The total PBP amount per enrolled participant was then distributed across seven scheduled study visits, proportionally to the work involved at each visit (Table 3). The Coordinating Center issued quarterly PBPs to the sites based on actual visits conducted, verified by data in the study’s electronic data capture system. Based on this model, a site would only receive its total approved 5-year budget if the targeted number of participants were enrolled and followed for the study duration.

**Table 1:** Suggested breakdown by the D2d Coordinating Center of personnel effort for a typical study site that is projected to enroll (i.e., randomize) and follow 125 participants

	Year 1 (%)	Year 2 (%)	Year 3 (%)	Year 4 (%)	Year 5 (%)
Principal investigator and co-investigator(s)	20	20	20	20	20
Research coordinator	100	100	100	75	75
Research assistant	50	100	100	75	75

Assumptions: 125 participants will be randomized by year 2. The bulk of participant follow-up is expected to occur in years 2–5

**Table 2:** Fundamental scheme of the hybrid financial management model utilized by D2d for a typical site that projected to enroll (i.e., randomize) 125 participants

	Year 1	Year 2	Year 3	Year 4	Year 5	Total
Personnel (effort and fringe benefits)	178,135	183,100	183,100	137,171	137,171	
Non-personnel	44,534	65,586	60,208	22,043	12,297	
Total proposed budget	222,669	248,686	243,308	159,214	149,468	1,023,345
Core						
Proposed Core^a^	89,068	91,550	91,550	68,586	68,586	409,339
Actual Core^b^	89,068	99,474	99,474	99,474	21,848	409,339
Performance-Based Payments						
PBP amount per enrolled participant: 4912						614,007

Amounts shown in US$; numbers include directs and 53% indirect rate. The scheme is flexible both from year to year and from site to site, while maintaining consistency

^a^Proposed Core was defined at the start of the study as 50% of annual personnel costs

^b^Actual Core varied from year to year (see text) while the total amount over 5 years did not exceed the total proposed Core (i.e., US$409,339)

**Table 3:** Scheme for Performance-Based Payments per participant visit for a typical D2d site that is projected to enroll (i.e., randomize) and follow 125 participants

	Per participant visit reimbursement	
Visit	% of total Performance-Based Payment amount	US$
Month 00 (randomization)	20	982
Month 6	6	295
Month 12	20	982
Month18	6	295
Month 24	20	982
Month 30	6	295
Month 36	22	1081
Total	100	4912^a^
Month 42^b^	6	295
Month 48^b^	22	1081

^a^Total site-specific Performance-Based Payment amount per enrolled participant who completes all seven scheduled study visits, month 00 to month 36 (see also text and Table 2). While the actual amount paid per participant visit varied by site (column 3), the percent of the total Performance-Based Payment amount allocated in each visit (column 2) was identical for all sites

^b^Visits beyond month 36 (month 42, etc.) are not included in the total site-specific Performance-Based Payment calculations because the average participant is expected to be followed for 36 months. A site that enrolls participants early in the recruitment period and follows these participants beyond month 36 will be reimbursed for additional visits (e.g., month 42 and month 48) in the same way as other similar visits (e.g., month 30 and month 36)

### Budget year 2: revisions to the model/addition of new sites

The model was revised in year 2 to better accommodate the sites’ evolving needs. The central components (Core and PBPs) were preserved, but Core was redefined as 40% of the total annual expenses (i.e., personnel effort *and* non-personnel costs) (Table 2). Quarterly PBPs continued to be made to the sites based on successful enrollment of participants and completion of follow-up visits.

Due to a delayed start of recruitment and lag in meeting enrollment targets, D2d added four new sites in year 2. The new sites submitted a standard 4-year NIH budget (grant years 2 to 5), and the revised methodology described above was used to determine Core and PBPs.

### Budget year 3 and beyond: further revisions to the model

Because recruitment continued through year 3 and part of year 4, the model was further revised. In years 3 and 4, sites received the same Core (in absolute US$) as in year 2 (Table 2). The model reduced the planned Core in year 5, such that the total Core received over 5 years was the same as the original proposed Core (Table 2). Sites were reminded that as the study progressed they would receive a larger proportion of their budget via PBPs.

### Transition to PBP-only for underperforming sites

The model implemented a uniform approach to manage underperforming sites. If the D2d leadership determined that there was potential for recruitment to improve at an underperforming site, the Coordinating Center continued to provide Core support for a limited “grace period” of 3, 4, or 6 months. During that time, the Coordinating Center worked closely with the underperforming site to establish new, realistic enrollment goals and supported the site to achieve the revised goals.

If a site did not meet its new target, site payments transitioned to PBP-only. The PBP-only alternative eliminated Core but increased the PBP amount per enrolled participant accordingly, so that the site was not penalized for continuing to enroll and follow participants.

### Primary outcome

The primary outcome of interest in relation to the hybrid model used by the D2d study was total expenses under this model compared to total expenses under a traditional model.

## Results

**Fig. 1:**
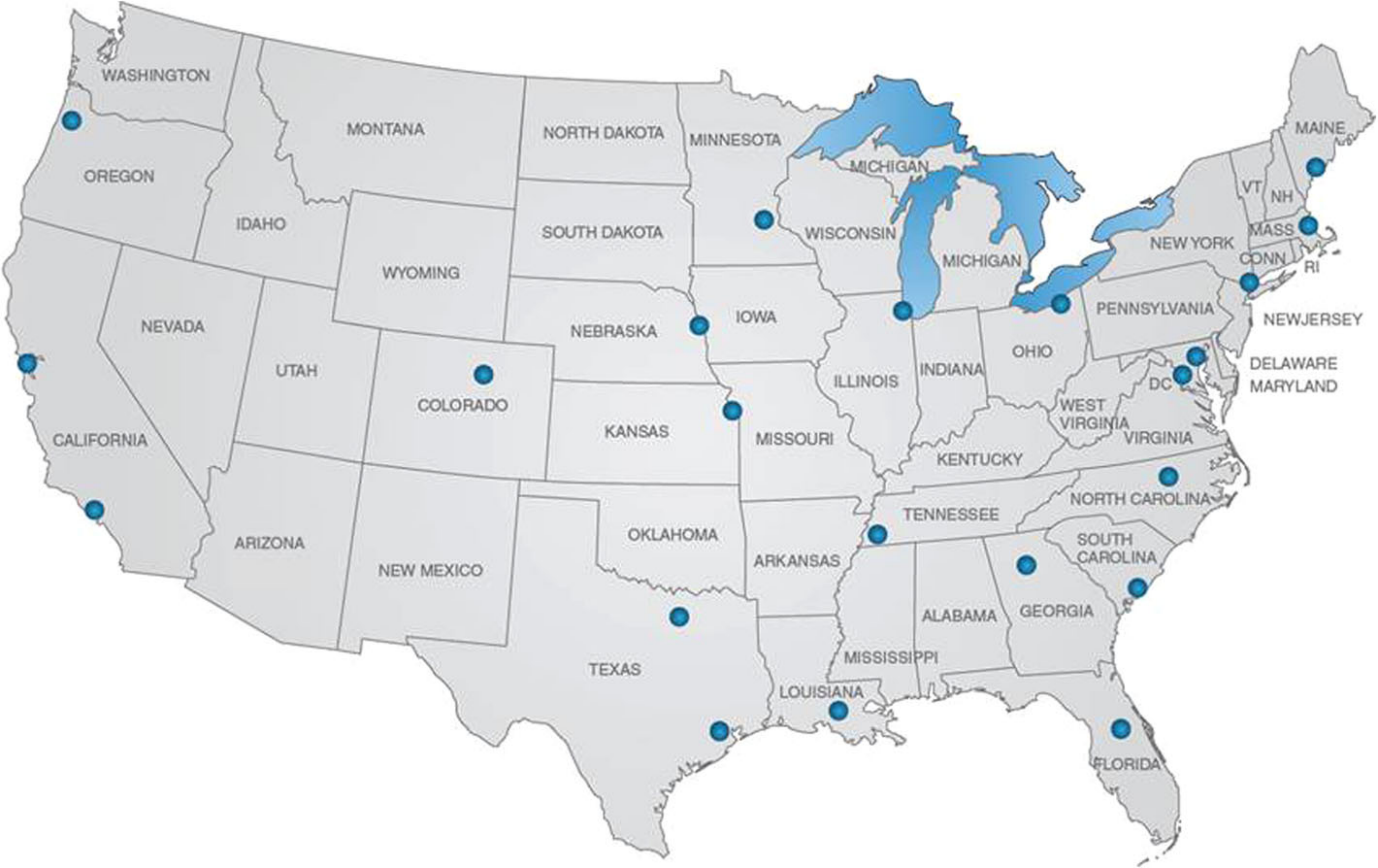
Location of collaborating clinical sites in the D2d study

Recruitment occurred from October 2013 through December 2016, and 2423 participants were randomized. The D2d clinical sites were located throughout the USA (Fig. 1) and included academic non-profit private medical centers, public medical centers, or Veterans Administration medical centers.

**Fig. 2:**
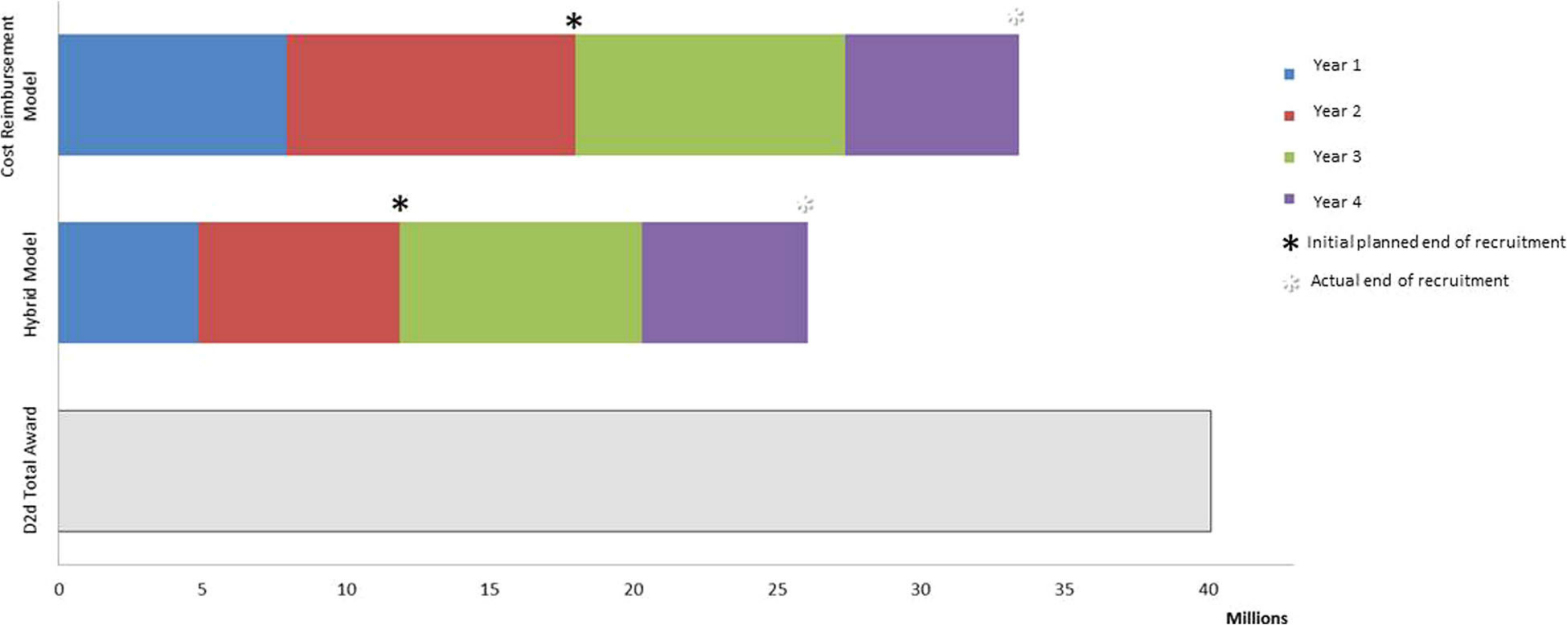
Comparison of the hybrid model used by D2d vs. a cost reimbursement subaward agreement for clinical sites. The cost reimbursement model includes the originally submitted budgets for sites (as cost reimbursement agreements) and actual expenses for non-site components (Coordinating Center, drug distribution, central lab) through the end of the recruitment phase without any budget reductions for underperformance. The hybrid model used by D2d includes actual spending (clinical sites and non-site components) through the end of the recruitment phase

The hybrid model used by D2d worked well to complete recruitment and have funds remaining to follow participants. Under a cost reimbursement model, an additional US$7.4M would have been needed to complete recruitment (Fig. 2). If D2d had issued cost reimbursement subaward agreements and sites invoiced for their entire budget, 83% of the award (US$33M) would have been spent compared to 65% of the award spent (US$26M) under the hybrid model used by D2d over the first 4 years of the trial (Fig. 2).

### Budget year 1: determination of Core vs. PBP value

In year 1, individual site budgets varied considerably (range US$3324 to US$8406 per participant to recruit and follow), largely due to varying salaries for research staff and fringe benefits (range US$38,027 to US$82,000 for research coordinators; US$25,094 to US$45,000 for research assistants; 15 to 41% for fringe benefits). These differences were attributed to regional differences (i.e., New York City vs. smaller cities in the Midwest), staff seniority, and expertise level.

In year 1, site activation and participant recruitment were slower than anticipated, which had a trickle-down effect on the model. Sites invoiced Core for 50% of actual personnel expenses, but could not fully recoup the remaining 50% through PBPs due to slow recruitment. At the end of year 1, study-wide recruitment was at 12% of the target (vs. 50% projected) and 12% of the total grant award was invested. Of the amount invested in year 1, 54% went to the clinical sites (46% as Core and 8% as PBPs) and 46% went to other components (Coordinating Center, central laboratory, and drug distribution). During year 1, two sites were discontinued, one due to personnel turnover and one for significant underperformance.

### Budget year 2: revisions to the model/addition of new sites

**Fig. 3:**
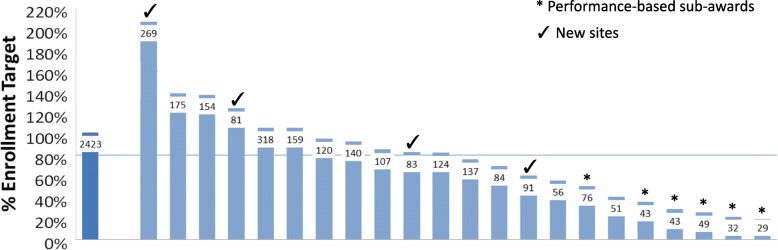
Enrollment in the D2d study. Enrollment is shown as percent of target, study-wide (dark blue bar) and for each clinical site (light blue bars). Horizontal line indicates the 80% mark of the enrollment target. Number of randomized participants is shown at the top of each bar. (√) indicates new sites added in year 2. (*) indicates sites that transitioned to a Performance-Based Payment-only arrangement

Revisions to the model and addition of new sites in year 2 were critical to the completion of recruitment. Revisions to the Core gave sites the flexibility to allocate Core support towards any study-related expense to meet site goals. At the end of recruitment, half of the sites met or exceeded the 80% mark of their recruitment target (Fig. 3). As in many large, multi-center trials, there was a wide range of recruitment success. Median enrollment at each site was 88 participants (range 29–318; 20 to 205% of the site target; Fig. 3). Three out of four new sites (75%) met or exceeded their enrollment target, vs. four out of 18 (22%) for the original sites. Of the D2d cohort, 20% was accounted for by the four new sites.

### Budget year 3 and beyond: further revisions to the model

As recruitment continued beyond year 2, into years 3 and 4, the changes in Core payments were critical to maintain recruitment activities. Increasing Core payments in years 3 and 4 and reducing Core payments in year 5 allowed recruitment to be completed within the original budget.

### Transition to PBP-only for underperforming sites

Transitioning underperforming sites to PBP-only agreements did not have an appreciable effect on recruitment or retention. Sites that transitioned to PBP-only agreements enrolled an average of 1.05 participant per month before the transition, compared to 1.35 participant per month after the transition. Sites that continued to receive Core and PBP randomized 2.5 participants per month. Sites receiving PBP-only completed 92% of all participant encounters vs. 95% for sites receiving Core and PBPs.

## Discussion

The hybrid financial management model used in the D2d study was designed to promote judicious investment of precious resources in a way that does not compromise recruitment and follow-up of participants. The fundamental principle behind the model is to allocate the total award earmarked for the sites proportional to their effort and results, while maintaining open-mindedness and flexibility to meet sites’ evolving needs. The methodology used to determine the Core and PBPs based on each site’s budget of what they expected their actual study costs over 5 years was novel. In the process, we learned several lessons (Table 4) that we discuss below.

**Table 4:** The D2d hybrid budget management model: lessons learned

• Encourage multi-disciplinary teamwork that engages the Principal Investigator, Coordinating Center, Research Administration at the prime, subaward institutions, and the funding agency.	
• Allocate resources based on results, not just effort.	
• Maintain open-mindedness and flexibility when addressing sites’ concerns.	
• Provide Core support and sufficient resources for study start-up.	
• Accept that sites will be discontinued and sites will be added.	
• Project that recruitment will take longer and the outcome event rate may be lower than predicted.	
• Reinforce the model’s principles to site staff.	

### Encourage multi-disciplinary teamwork

In D2d, the study’s Principal Investigator, Coordinating Center, Research Administration at the prime grantee institution, and the funding agency worked collaboratively to design and employ a principles-based financial management model to ensure appropriate stewardship of the available funds. The model aimed for consistency while recognizing sites’ unique circumstances. At least in part, the hybrid model used by D2d worked well because people from different disciplines established a close and dynamic collaboration and decisions were made in a cooperative fashion with the clinical sites.

### Allocate resources based on results, not just effort

Although effort and results often correlate, the association is far from perfect, as effort may be exerted inefficiently or ineffectively. For example, bulk mailing of non-targeted letters is an inefficient recruitment method compared to an electronic health record (EHR)-based recruitment approach, especially for a trial with laboratory-based inclusion criteria. To allocate resources based on effort and results, the model established expectations in terms of recruitment and retention targets and timelines. Site recruitment was monitored weekly by the study leadership, and the model encouraged sites to abandon inefficient approaches. The bulk of the funds provided to the sites matched recruitment and retention results, especially as the study progressed.

At the outset, recognizing that few sites would reach their goal, the Coordinating Center estimated that the average site would enroll about 80% of its projected target, while a few sites would exceed their target. The prediction was correct, as exactly half of the sites met or exceeded the 80% mark at the end of the recruitment period. However, the range of recruitment success (20 to 205% of the stated goal) was larger than anticipated. Therefore, flexibility to divert funds away from underperforming sites to match needs of well-performing sites was a key component of the model.

### Maintain open-mindedness and flexibility when addressing sites’ concerns

Although the hybrid payment model resulted in a more efficient investment of research funds in direct proportion to results, many sites faced budget deficiencies due to underperformance and unanticipated issues, including increases in fringe rate, facilities and administrative rate, and other expenses not accounted for in their original budget. Sites also did not fully appreciate that they would receive their entire approved 5-year budget only if they fully met their enrollment and retention goals. For example, if a site reached 80% of its enrollment target and successfully completed all follow-up visits, its PBPs would be reduced proportionally by 20%.

Recognizing sites’ unique circumstance is important in large, multi-center trials and decisions need to be made in a consistent manner. The D2d leadership kept an open mind throughout the recruitment phase—the most variable phase of a clinical trial—and worked individually with sites, recognizing the uniqueness of the challenges faced by each, and strived to be flexible. For example, in each year during the recruitment phase, the D2d leadership used a uniform approach to adjust the Core to better accommodate sites’ needs. For outlier sites, the D2d leadership re-established expectations and adjusted the budget as needed. When faced with the potential of a site withdrawing from the study due to financial issues, the D2d leadership worked with the site to modify recruitment goals and its budget, while staying within the framework of the model.

### Provide Core support and sufficient resources for study start-up

A key concept of the hybrid model used by D2d is to provide Core funds for study start-up (e.g., IRB submission, study setup) and partial salary support (e.g., pre-screening of participants, participation in committees). While a PBP-only model is conservative and advantageous to the total study budget, under such model, sites may be reluctant to invest in the initial effort, including exploring innovative recruitment methods. Study-wide, providing Core funds may be a risky approach if many sites underperform. In D2d, providing Core for a proportion of annual personnel effort only did not work. Redefining the Core as 40% of the annual total budget (including both personnel and non-personnel) worked well and allowed sites to overcome administrative concerns. The Core component proved to be critical to the success of D2d as it allowed sites to invest in the successful launch of the trial.

### Accept that sites will be discontinued and sites will be added

Despite extensive experience and initial enthusiasm, a few sites will never get off the ground and many will underperform. There are many reasons that can prevent sites from reaching their full potential, including natural disasters, research staff relocation or retirement, illness affecting key personnel, and shifting priorities at the site. It is important for the leadership team to identify sites that have the potential to improve with appropriate guidance and sites that need to be discontinued. The decision to discontinue a site is exceedingly difficult because of financial, human (personnel), academic (collegial), and emotional implications. There are ethical concerns in relation to the investigators’ duty to enrolled participants. However, timely separation is necessary as delay only utilizes more funds and makes the discontinuation even more problematic, especially when many participants need to be administratively withdrawn. Discontinuation of sites needs to follow a consistent process, and the decision needs to be unanimous by the study leadership.

It is certain that not all original sites will perform as projected; therefore, adding new sites should be anticipated and planned. Adding sites while the trial is ongoing is logistically challenging; however, the leadership, based on accumulated study-specific experience, is better prepared to evaluate and select new sites whose strengths match the study’s needs. The addition of new sites proved critical to the success of D2d.

### Recruitment will take longer and the outcome event rate may be lower than projected

It is rare for large trials to complete recruitment within the original timeline. In D2d, the recruitment period was projected to last 2 years; however, despite the sites’ best efforts, including adoption of a very successful electronic health record-based recruitment approach, enrollment was slower than projected. Furthermore, the rate of occurrence of the primary outcome can be lower than projected [8], which may require study extension, thereby adding strain to the budget. The hybrid model used by D2d conserved funds to ensure completion of recruitment while conserving funds to continue following participants. If D2d had issued cost reimbursement agreements and sites invoiced for their entire budget without any modifications for underperformance, nearly the entire grant award would have been spent at the completion of recruitment.

### Reinforce the model’s principles to site staff

A hybrid model can be new to investigators and research administration staff; therefore, it is important to reinforce the principles of the model throughout the study. When executing the original subawards, the Coordinating Center provided a written document that described the model. As the model was enhanced each year, the D2d leadership maintained open communication with sites to optimize understanding and application of the model, especially given turnover of site research administrative staff.

### Limitations

The model worked for D2d, which is a focused and highly structured trial with laboratory-based eligibility criteria and outcomes. The model may be more difficult to adapt in a more complicated trial with multiple outcomes. Other groups are exploring different methods to minimize costs of clinical trials, such as use of mobile technology or electronic medical records [9, 10]. Additionally, the model was applied in a trial supported by the NIH, and results may not apply to non-NIH trials or those outside of the USA.

## Conclusions

The NIH-supported D2d clinical trial employed a hybrid financial management model, comprised of two components (Core and Performance-Based Payments) to manage the study’s budget in a dynamic and creative way to effectively invest finite resources. The development and employment of the model is the result of a cooperative effort between Research Administration at the prime grantee institution, Principal Investigator, Coordinating Center, and the primary funding agency. Anticipating a delay in recruitment, the model continuously evaluated sites’ needs and re-allocated resources to meet the study enrollment goal and succeeded in managing the study’s funds during the extended recruitment period in a way that did not compromise the trial’s scientific integrity.

## Abbreviations

D2d: Vitamin D and Type 2 Diabetes
NIDDK: National Institute of Diabetes and Digestive and Kidney Diseases
NIH: National Institutes of Health
PBPs: Performance-Based Payments
